# Supplementary material for: Evolution of DNA methylome from precancerous lesions to invasive lung adenocarcinomas
Source: Nat Commun. 2021 Jan 29;12:687. doi: 10.1038/s41467-021-20907-z (PMC7846738; doi:10.1038/s41467-021-20907-z)
Supplement: Supplementary file 2 — Description of Additional Supplementary Files [file 41467_2021_20907_MOESM2_ESM.pdf]

Description of Additional Supplementary Files

File Name: Supplementary Data 1
Description: Patient clinical characteristics

File Name: Supplementary Data 2
Description: Summary of clinical characteristics associated with different specimens

File Name: Supplementary Data 3
Description: DNA motifs associated with DMRs enriched in lung ADC and its precursors of different stages

File Name: Supplementary Data 4
Description: DNA motifs significantly matched to known transcription factor (TF) binding sites

File Name: Supplementary Data 5
Description: Transcription factor binding sites and histone marks significantly enriched in chromosomal regions with eloci in each stage

File Name: Supplementary Data 6
Description: Clinical, genomic and epigenomic features in IPNs with more than 4 multi-region samples

File Name: Supplementary Data 7
Description: Putative tumor suppressor genes showing evidence of convergent genomic and epigenomic evolution
