# Supplementary material for: Evolution of DNA methylome from precancerous lesions to invasive lung adenocarcinomas
Source: Nat Commun. 2021 Jan 29;12:687. doi: 10.1038/s41467-021-20907-z (PMC7846738; doi:10.1038/s41467-021-20907-z)
Supplement: Supplementary file 4 — Reporting Summary [file 41467_2021_20907_MOESM4_ESM.pdf]

nature research

Corresponding author(s): Jianjun Zhang

Last updated by author(s): Nov 28, 2020

Reporting Summary

Nature Research wishes to improve the reproducibility of the work that we publish. This form provides structure for consistency and transparency in reporting. For further information on Nature Research policies, see our Editorial Policies and the Editorial Policy Checklist.

Statistics

For all statistical analyses, confirm that the following items are present in the figure legend, table legend, main text, or Methods section.

n/a	Confirmed	
☐	☒	The exact sample size (*n*) for each experimental group/condition, given as a discrete number and unit of measurement
☐	☒	A statement on whether measurements were taken from distinct samples or whether the same sample was measured repeatedly
☐	☒	The statistical test(s) used AND whether they are one- or two-sided<br>*Only common tests should be described solely by name; describe more complex techniques in the Methods section.*
☐	☒	A description of all covariates tested
☐	☒	A description of any assumptions or corrections, such as tests of normality and adjustment for multiple comparisons
☐	☒	A full description of the statistical parameters including central tendency (e.g. means) or other basic estimates (e.g. regression coefficient) AND variation (e.g. standard deviation) or associated estimates of uncertainty (e.g. confidence intervals)
☐	☒	For null hypothesis testing, the test statistic (e.g. *F*, *t*, *r*) with confidence intervals, effect sizes, degrees of freedom and *P* value noted<br>*Give P values as exact values whenever suitable.*
☒	☐	For Bayesian analysis, information on the choice of priors and Markov chain Monte Carlo settings
☐	☒	For hierarchical and complex designs, identification of the appropriate level for tests and full reporting of outcomes
☐	☒	Estimates of effect sizes (e.g. Cohen's *d*, Pearson's *r*), indicating how they were calculated

Our web collection on statistics for biologists contains articles on many of the points above.

Software and code

Policy information about availability of computer code

Data collection	no software was used for data collection, we only performed analysis on 53 resected pulmonary nodules and paired normal lung tissues from 39 patients, who underwent surgical resection.
Data analysis	TrimGalore v.0.4.3 was used to trim Illumina adapter sequences; Bismark v.0.18.1 and bowtie2 v.2.2.3 was used to align the trimmed reads; FastQC v.0.11.7 was used for quality control. The DNA methylation levels for individual CpGs were calculated using methyKit (v.1.16.0). The CpG sites or DMRs for genomic location was annotated using R package “ChIPSeeker” (v.1.26.0) and toolkit “genomation”(v.1.22.0). DMRs were detected using “DMR caller”(v.1.22.0) R package. ChromHMM v.1.21 is applied to partition of functional genomic regions based on chromatin states model. MethylSeekR (v.1.30.0) package is used to identify PMDs. The de novo methylated DNA motifs were identified using mEpigram (v.0.07), Tomtom (v.5.2.0) tool from MEME suite was used to select significantly enriched methylated DNA motifs to the database of known transcript factors (HOCOMOCO_v11). Epiallele shift was identified by “methclone” (v.0.1). Locus Overlap Analysis was performed using Locus Overlap Analysis (LOLA). The “ape” (v.5.4.1) package was used to build phylogeny trees. Locus Overlap Analysis (LOLA) (v.1.20.0) was used to perform Locus Overlap Analysis. ImmuCellAI was applied to infer immune cell components for RNAseq data. “MethylCIBERSORT” (v.0.2.0) package was used to estimate immune cell components for RRBS data. “stat_summary” and “geom_violin” function is from ggplot2 (v.0.9.1).

For manuscripts utilizing custom algorithms or software that are central to the research but not yet described in published literature, software must be made available to editors and reviewers. We strongly encourage code deposition in a community repository (e.g. GitHub). See the Nature Research guidelines for submitting code & software for further information.

Data

Policy information about availability of data

All manuscripts must include a data availability statement. This statement should provide the following information, where applicable:

- Accession codes, unique identifiers, or web links for publicly available datasets
- A list of figures that have associated raw data
- A description of any restrictions on data availability

WES dataset is available from EGA: EGAD00001004960 (https://www.ebi.ac.uk/ega/datasets/EGAD00001004960); RRBS dataset is available from EGA: EGAS00001004610 (https://www.ebi.ac.uk/ega/studies/EGAS00001004610); GSM983647 was retrieved from GEO (https://www.ncbi.nlm.nih.gov/geo/query/acc.cgi?acc=GSM983647).EGAS00001004006 was downloaded from EGA (https://www.ebi.ac.uk/ega/studies/EGAS00001004006). EGAD00001002492 (https://www.ebi.ac.uk/ega/datasets/EGAD00001002492) and EGAD00001002486 (https://www.ebi.ac.uk/ega/datasets/EGAD00001002486) was downloaded from EGA, respectively. All other data may be found within the main manuscript or supplementary Information or available from the authors upon request.

Field-specific reporting

Please select the one below that is the best fit for your research. If you are not sure, read the appropriate sections before making your selection.

☒ Life sciences ☐ Behavioural & social sciences ☐ Ecological, evolutionary & environmental sciences

For a reference copy of the document with all sections, see nature.com/documents/nr-reporting-summary-flat.pdf

Life sciences study design

All studies must disclose on these points even when the disclosure is negative.

Sample size	Sample size was not determined before the study, rather based on sample availability. Because surgical resection is not the standard of care, there has been scarcity of resected specimens from lung adenocarcinoma precursors. The specimens were collected through international collaborations. All available samples were subjected to the analysis. In addition, the methylation aberrations in development and progression of lung precancers were unknown, therefore it was impossible to determine the power prior to RRBS profiling.
Data exclusions	No data were excluded from the analyses, as all samples and RRBS library was already subjected to rigorous QC by the sequence core.
Replication	Due to scarcity of resected specimens from lung adenocarcinoma precursors, all available samples were subjected to the analysis. Replication was not feasible.
Randomization	This is not a study involving clinical trial, randomization does not apply to this study. Due to scarcity of resected specimens from lung adenocarcinoma precursors, all available samples were subjected to RRBS profiling and the corresponding analysis.
Blinding	This is not a study involving clinical trial, blinding does not apply to this study.

Reporting for specific materials, systems and methods

We require information from authors about some types of materials, experimental systems and methods used in many studies. Here, indicate whether each material, system or method listed is relevant to your study. If you are not sure if a list item applies to your research, read the appropriate section before selecting a response.

Materials & experimental systems

n/a	Involved in the study	
☒	☐	Antibodies
☒	☐	Eukaryotic cell lines
☒	☐	Palaeontology and archaeology
☒	☐	Animals and other organisms
☐	☒	Human research participants
☒	☐	Clinical data
☒	☐	Dual use research of concern

Methods

n/a	Involved in the study	
☒	☐	ChIP-seq
☒	☐	Flow cytometry
☒	☐	MRI-based neuroimaging

Human research participants

Policy information about studies involving human research participants

Population characteristics	All patients are from China and Japan, with median age of 69.8 (range 44-80.5) and the ratio of male to female is 22:17. All patients presented as pulmonary nodules and treated with upfront surgical resection. None of the patients received neoadjuvant treatment.
Recruitment	No patients were recruited specifically for this study. All patients were treated as standard of care and surgical specimens were analyzed. No self-selection process is involved.
Ethics oversight	Written informed consent was obtained from all patients involved. The study was approved by institutional review board (IRB) from Nagasaki University Hospital, Zhejiang Cancer Hospital and MD Anderson Cancer Center. This study is compliant with the "Guidance of the Ministry of Science and Technology (MOST) for the Review and Approval of Human Genetic Resources", which requires formal approval for the export of human genetic material or data from China.

Note that full information on the approval of the study protocol must also be provided in the manuscript.
